# Garnet-Based All-Ceramic Lithium Battery Enabled by Li_2.985_B_0.005_OCl Solder

**DOI:** 10.1016/j.isci.2020.101071

**Published:** 2020-04-18

**Authors:** Wuliang Feng, Zhengzhe Lai, Xiaoli Dong, Panlong Li, Yonggang Wang, Yongyao Xia

**Affiliations:** 1 Department of Chemistry and Shanghai Key Laboratory of Molecular Catalysis and Innovative Materials, Institute of New Energy, Fudan University, Shanghai 200433, China

**Keywords:** Energy Materials, Energy Storage, Mechanical Property

## Abstract

Garnet-based bulk-type all-ceramic lithium battery (ACLB) is considered to be highly safe, but its electrochemical performance is severely hindered by the huge cathode/electrolyte interfacial resistance. Here, we demonstrate an *in situ* coated Li_2.985_B_0.005_OCl as sintering solder, which is uniformly coated on both LiCoO_2_ and Li_7_La_3_Zr_2_O_12_. With the low melting point (267°C) and high ionic conductivity (6.8 × 10^−5^ S cm^−1^), the Li_2.985_B_0.005_OCl solder not only restricts La/Co interdiffusion, but also provides fast Li^+^ transportation in the cathode. A low cathode/electrolyte interfacial resistance (386 Ω cm^2^) is realized owing to the densification of the ACLB by hot-press sintering. The strain/stress of the LiCoO_2_ is also released by the small elasticity modulus of Li_2.985_B_0.005_OCl, leading to a superior cycling stability. The study sheds light on the design of advanced garnet-based bulk-type ACLB by exploring proper solders with higher ionic conductivity, lower melting point, and smaller elasticity modulus.

## Highlights

• Hot-press sintering is developed in densifying the all-ceramic lithium battery
• Li_2.985_B_0.005_OCl displays a high ionic conductivity and a low melting point
• Li_2.985_B_0.005_OCl layer is *in situ* coated on both garnet and LiCoO_2_ uniformly
• The strain/stress effects of the LiCoO_2_ is released during charge/discharge

## Graphical Abstract

**Figure:**
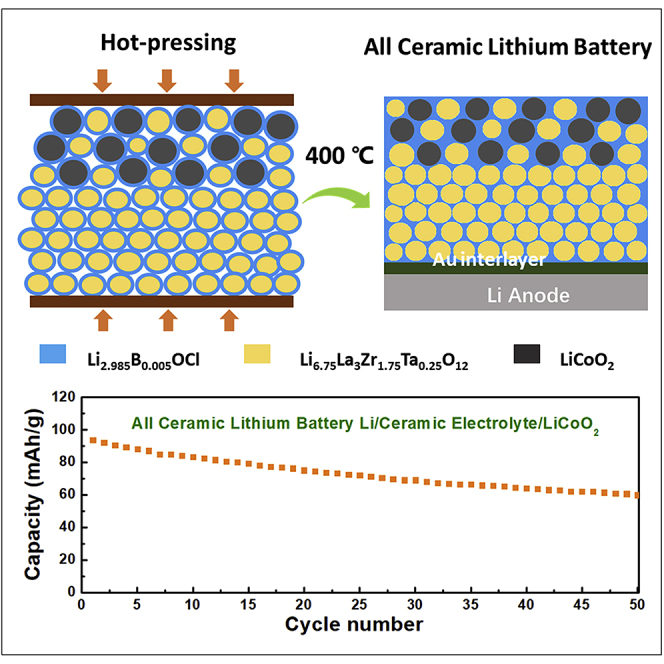

## Introduction

The all-ceramic lithium battery (ACLB) is regarded as the ultimate goal to exclude the safety concerns for Li-ion battery (Janek and Zeier, 2016, Zhang et al., 2018a, Zhang et al., 2018b). To successfully achieve an ACLB with satisfied electrochemical performance, electrolyte/electrode interfacial problem is the most changing part rather than the ionic conductivity of the solid-state electrolytes (SSEs) (Jena, et al., 2018). Sulfide-based SSEs are soft enough to reduce the interfacial resistance just by high-pressure treatment. However, there remains a poor electrochemical stability against electrodes and a risk of toxic H_2_S release (Han et al., 2016, Wenzel et al., 2016, Matthew et al., 2019, Wu et al., 2018). Very recently, halide-based SSEs Li_3_MCl_6_ (M = In, Y, Er) with ionic conductivity over 1 mS cm^−1^ have been developed, and the Young's modulus is also low enough to enable its bulk ACLB assembly without any heat treatment (Li et al., 2019a, Li et al., 2019b, Schlem et al., 2019). Unfortunately, the halide SSEs are hygroscopic and also unstable with metal Li (Asano et al., 2018). Among all of the SSEs, garnet-type SSEs Li_7_La_3_Zr_2_O_12_ (LLZO) exhibit the widest electrochemical window against Li and a variety of cathodes, making it an attractive candidate for bulk-type ACLB (Zhao et al., 2019, Liu et al., 2018a, Liu et al., 2018b, Li et al., 2017).

Regardless of the lithiophobic layers on the surface, which lead to large Li/LLZO interfacial resistance, the issue has already been effectively solved by surface polishing (Sharafi et al., 2017, Wu et al., 2019) and interlayers (Han et al., 2017, Alexander et al., 2018, Feng et al., 2019, Huo et al., 2019, Fu et al., 2019) or composite anodes integrating (Lu et al., 2018, Wang et al., 2018). The area-specific resistance (ASR) has been reduced by three orders of magnitude, and Li dendrite can also be suppressed owing to the moderated Li stripping and plating environment (Li et al., 2018).

By contrast, there are few studies available in garnet-based bulk-type ACLB since the cathode/LLZO interface is the main obstacle (Park et al., 2016). The circumstance of cathode/LLZO interface is more complex than that of Li/LLZO, which includes (1) huge interfacial resistance due to the lack of contact between active materials and LLZO (Liu et al., 2017), (2) the formation of highly ionic resistive phase during sintering (Kim et al., 2011, Zhang et al., 2018a, Zhang et al., 2018b), (3) and the cracks at the interface due to the volume change of the active materials (Okumura et al., 2016). Explorations have been done on enlarging the contact area of LLZO with active materials by building a 3D LLZO surface (Broek et al., 2016, Hänsel et al., 2016, Shoji et al., 2016), whereas the discharge capacities are still far from satisfied. Owing to the rigid character of the ceramic-to-ceramic interface, high-temperature sintering is an indispensable step to improve the contact of the active materials with LLZO. However, there remains a risk of forming Li^+^-resistive interdiffusion phase of LaXO_3_ (X = Ni, Co, Mn) even at the temperature as low as 500°C (Vardar et al., 2018). To simultaneously improve the contact of the active materials with LLZO and suppress the interdiffusion phase, solders such as Li_3_BO_3_, Li_2.3_C_0.7_B_0.3_O_3_, and Li_2.2_C_0.8_B_0.2_O_3_ have been applied to the ACLBs (Park et al., 2016, Ohta et al., 2013, Ohta et al., 2014, Han et al., 2018, Okumura et al., 2016). In addition, 0.44LiBO_2_.0.56LiF and Li_3_BO_3_.ITO have also been introduced to synthesize the all-ceramic cathodes (Chen et al., 2014, Liu et al., 2018a, Liu et al., 2018b). However, the ionic conductivities of the solders are in the 10^−6^ S cm^−1^ scale or even lower, which can be a constraint factor for Li^+^ transport. Moreover, the melting point of the solder is much higher than 500°C, which leads to even higher sintering temperature. Thus, the cathode still suffers from forming interdiffusion phase once there remains a direct contact of the active materials with LLZO.

In the present work, we proposed a trivalent element doped Li_2.985_B_0.005_OCl anti-perovskite as an effective solder for the ACLB, which demonstrated not only an ionic conductivity more than one order of magnitude higher than the present candidates (6.8× 10^−5^ S cm^−1^), but also a melting point as low as 267°C. A novel alkaline aqueous solution-based *in situ* coating of Li_2.985_B_0.005_OCl on both Li_6.75_La_3_Zr_1.75_Ta_0.25_O_12_ (LLZTO) and LiCoO_2_ (LCO) was introduced, in which a Li_2.985_B_0.005_OCl layer was uniformly coated on the particle surface. Both Li^+^/H^+^ exchange suppression in LLZTO and LCO/LLZTO thorough segregation were realized. The critical current density (CCD) of the LLZTO@Li_2.985_B_0.005_OCl composited electrolyte (CE) was improved from 0.6 to 0.8 mA/cm^2^ owing to the reduced electronic conductivity at the grain boundary. The ACLB was assembled in a simple way by co-hot-press sintering of the CE and the cathode at 400°C, which displayed a smaller impedance than the traditional cold-press sintered counterpart. Attributing the lower elasticity modulus, Li_2.985_B_0.005_OCl is soft enough to release more strain/stress effects of the LCO during charge/discharge, and the Li_2.985_B_0.005_OCl soldered ACLB demonstrated higher capacity and cycling stability than the Li_3_BO_3_ and LiF soldered counterparts.

## Results and Discussion

### Li_2.985_B_0.005_OCl *In Situ* Coating and the ACLB Assembling

**Figure 1:**
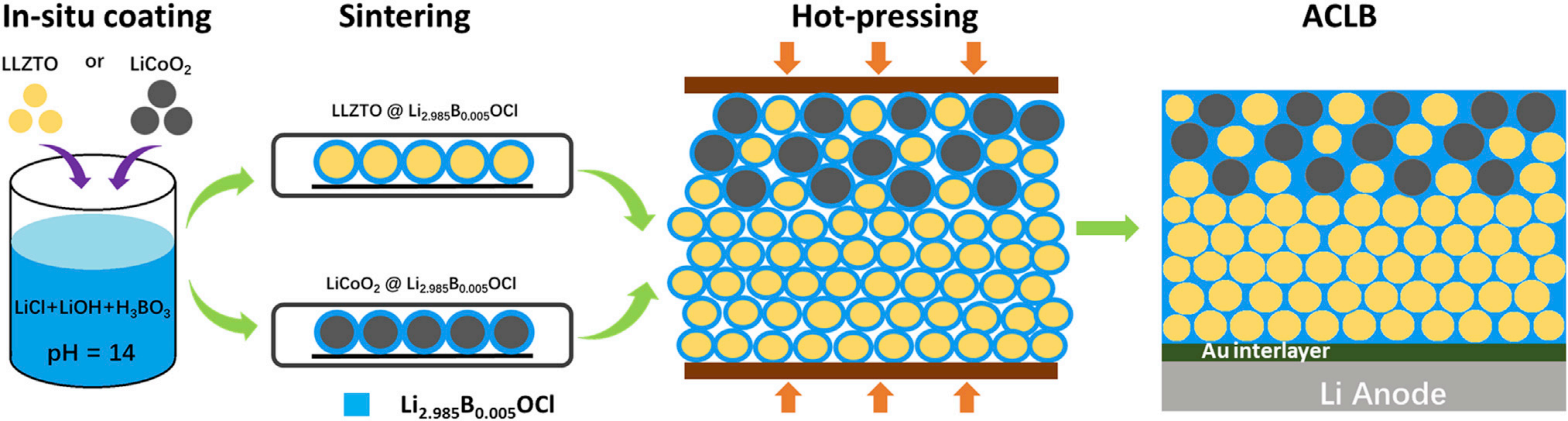
Schematic Diagram of Surface Coating of Li_2.985_B_0.005_OCl and Assembling of ACLB

As illustrated in Figure 1, Li_2.985_B_0.005_OCl was *in situ* coated on LCO and LLZTO in alkaline aqueous solution. Li^+^/H^+^ exchange reaction in LLZTO can be avoided by increasing the pH value to 14 by adjusting the stoichiometric amounts of the starting materials (LiCl, LiOH, and H_3_BO_3_), and the concentration of the LiOH should be over 1 M to ensure the required pH value. The reaction equation based on the starting material is $\text{Li}+1.985\text{ LiOH}+0.005{\text{ H}}_{3}{\text{BO}}_{3\;}\to {\text{ Li}}_{2.985}{\text{B}}_{0.005}\text{OCl}+{\text{ H}}_{2}\text{O }$. The coated LLZTO is in cubic phase and no La_2_Zr_2_O_7_ lithium-devoid phases can be indexed, indicating the successfully suppressed Li^+^/H^+^ exchange (Figure S1A). To acquire the Li_2.985_B_0.005_OCl anti-perovskite on the surface, the coated LCO and LLZTO were sintered at 350°C under vacuum. As the X-ray photoelectron spectra shown in Figure S2, Cl-O bonding can be found in both Cl 2p and O 1s core levels, indicating the formation of the Li_2.985_B_0.005_OCl anti-perovskite. In regard to the ACLB assembly, considering that the conventional synthesized LLZTO pellet (sintered over 1,000°C) is fragile and unable to sustain any high pressure, the CE was co-hot-pressed together with the cathode at 400°C, to simultaneously melt the Li_2.985_B_0.005_OCl solder in cathode and the CE. Finally, Au thin film was deposited on one side of the CE to improve the wettability of molten Li and reduce the interfacial resistance.

### Characterization of the Anti-perovskite Electrolytes

**Figure 2:**
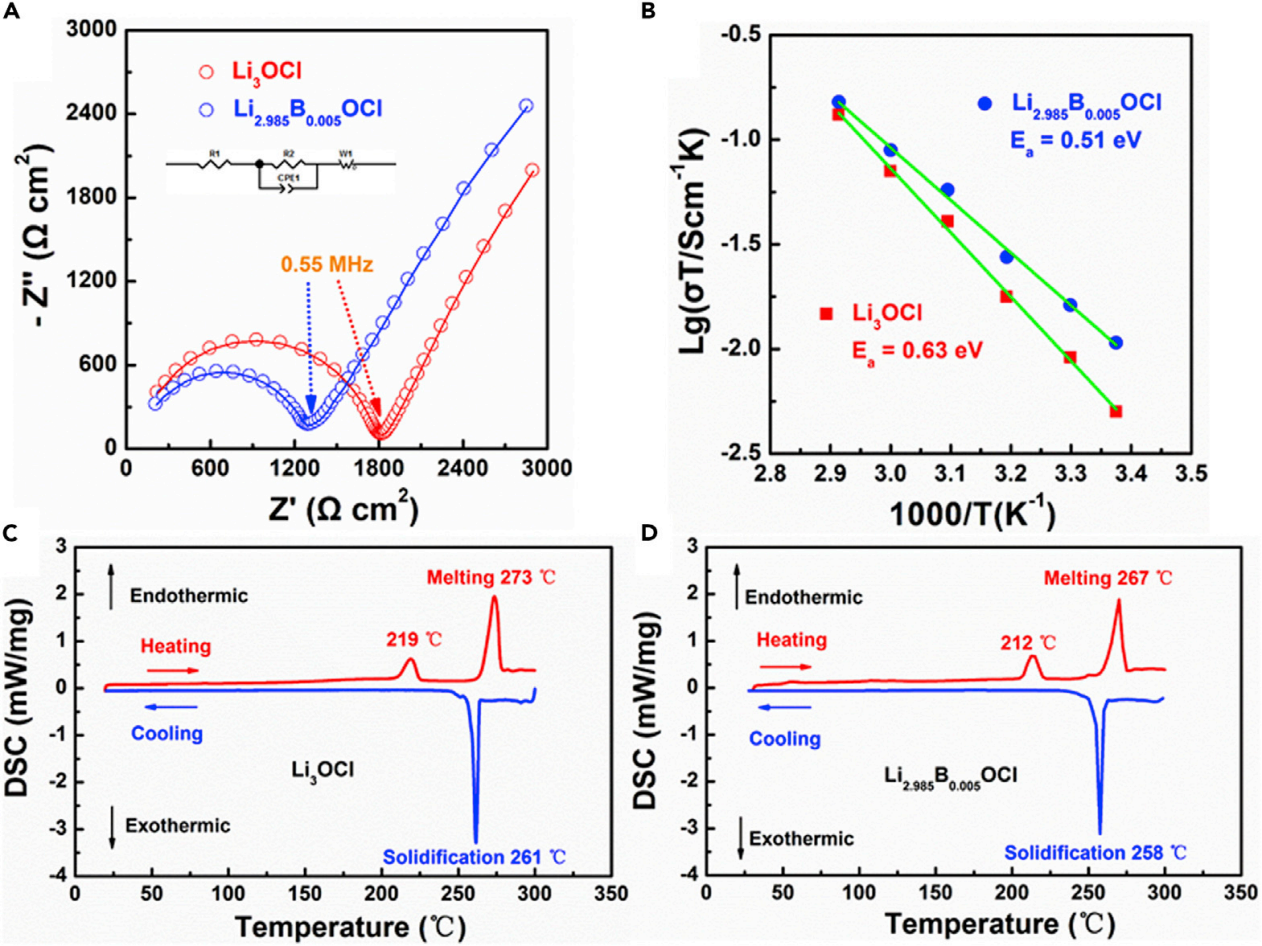
Characterization of the Anti-Perovskites (A) Nyquist plot of the anti-perovskites without and with B^3+^ doping. (B) Arrhenius plot of the anti-perovskites without and with B^3+^ doping. (C and D) DSC data of the (C) Li_3_OCl and (D) Li_2.985_B_0.005_OCl electrolytes.

Based on the conventional Li_3_OCl anti-perovskite electrolyte, trivalent element B^3+^ was introduced to further increase the ionic conductivity of the solder. According to the EIS profiles in Figure 2A, the ionic conductivity of Li_3_OCl and Li_2.985_B_0.005_OCl are 4.9 × 10^−5^ and 6.8 × 10^−5^ S cm^−1^ respectively. As shown in the Arrhenius plot (Figure 2B), the activation energy for Li^+^ diffusion was decreased from 0.63 to 0.51 eV by the vacancy creation, which led to the faster ionic transportation. Herein the molecular formula (i.e., Li_2.985_B_0.005_OCl) is only used to briefly describe the material composition. As the prepared material shows the amorphous characteristics with a low diffraction peak density in XRD pattern (Figure S1B), at the current stage, it is very hard to confirm the crystalline framework of the B-modified Li_3_ClO. However, it should be noted that B-doping is not the key point of the present work, and thus we do not give further discussion on this point. Different kinds of alkali metal cations have been doped to improve the ionic conductivity of the anti-perovskite by creating more vacancies in the cation sublattice (Braga et al., 2014). With the doping of a higher-valent cation B^3+^, more vacancies and Li^+^ transport paths can be potentially created by coordinating the charge balance. The structural changes and melting and solidification points are characterized by differential scanning calorimetry (DSC). The anti-perovskite without dopant shows a phase transition peak at 219°C, a melting peak at 273°C, and a solidification peak at 261°C (Figure 2C). With B^3+^ doping, the phase transition, melting, and solidification points are 7°C, 6°C, and 3°C lower than that of Li_3_OCl (Figure 2D). The melting and solidification points are presently the lowest among all of the solder for garnet-based ACLB and are essential to reduce the heat treatment from conventional 700°C to only 400°C.

### Characterization of the *In Situ* Coating of Li_2.985_B_0.005_OCl

**Figure 3:**
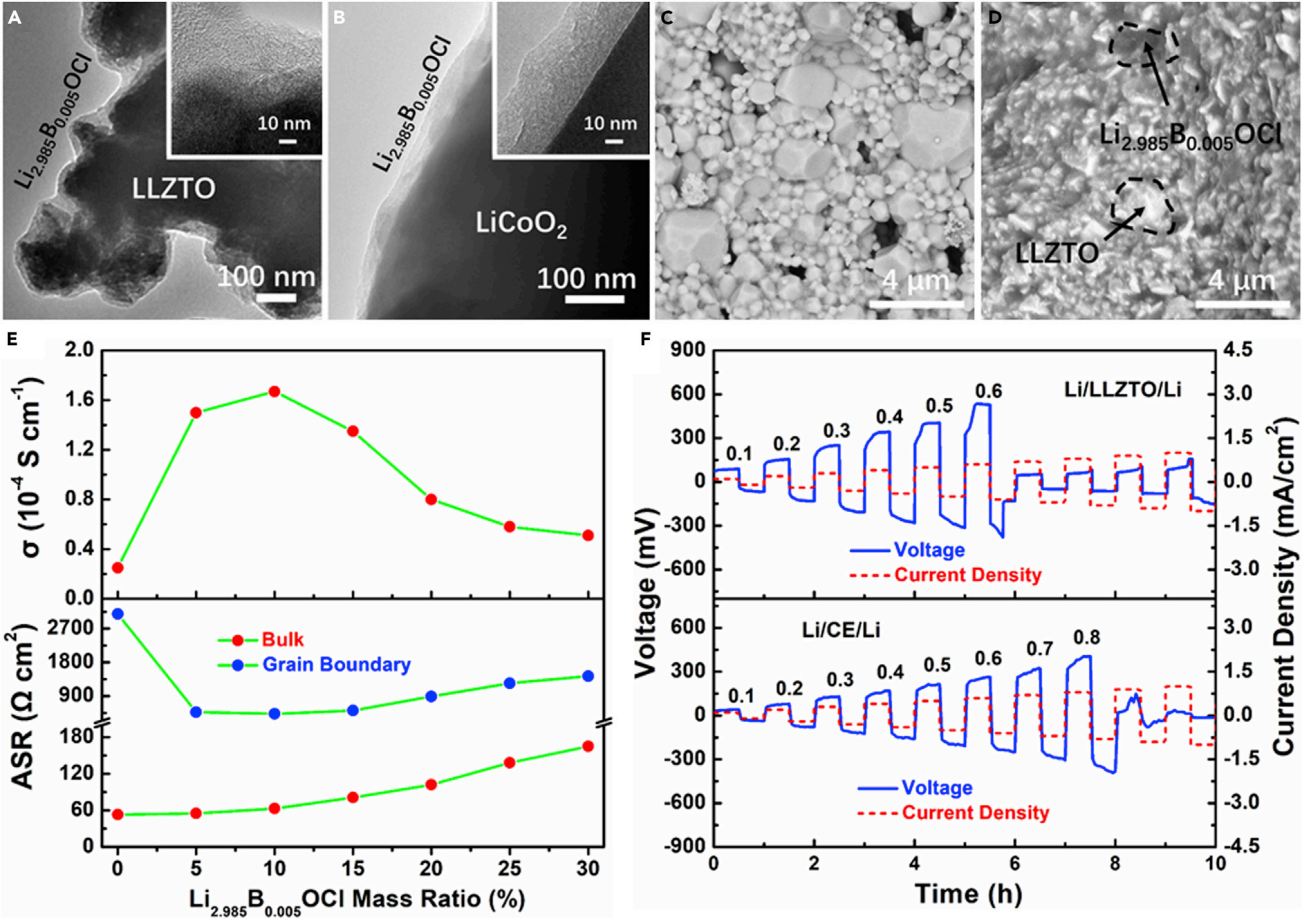
Characterization of the Composited Electrolyte (A and B) TEM images of the coated (A) LLZTO and (B) LCO particles. (C and D) Cross-sectional SEM images of the (C) LLZTO and (D) LLZTO@Li_2.985_B_0.005_OCl electrolytes being hot pressed at 400°C. (E) Li^+^ conductivity, ASR of the bulk and grain boundary as a function of Li_2.985_B_0.005_OCl mass ratio. (F) CCD testing of the Li/LLZTO/Li and Li/CE/Li symmetric cells.

As the transmission electron microscopy (TEM) image illustrated in Figures 3A and 3B, the *in situ* coated Li_2.985_B_0.005_OCl has covered all of the surface and every corner of LLZTO and LCO particles, which is vital to realize the thoroughly separated LLZTO and LCO. The mapping of Cl and Zr in Figures S3A–S3C demonstrated that the Li_2.985_B_0.005_OCl is evenly coated. Comparing with Figures 3A and 3B, TEM of the pristine LLZTO (Figure S3D) and LCO (Figure S3F) shows that the particles are exposed without the coating layer. The cross-sectional scanning electron microscopy (SEM) images in Figures 3C and 3D compare the porosity of the pristine LLZTO and the CE that were treated at 400°C. Evident holes can be found in the pristine LLZTO, whereas the Li_2.985_B_0.005_OCl soldered LLZTO is much denser and no holes can be found. To study the influence of the Li_2.985_B_0.005_OCl mass ratio on the electrochemical performance of the CE, the staring materials of LiCl, LiOH, and H_3_BO_3_ were adjusted to different concentrations to get the CE with Li_2.985_B_0.005_ClO mass ratio from 5% to 30%. The EIS profiles of the CE with different Li_2.985_B_0.005_OCl mass ratio are demonstrated in Figure S4 and the fitted results are displayed in Table S1. Owing to the large amounts of voids, the pristine LLZTO shows the least ionic conductivity. With the soldering of Li_2.985_B_0.005_OCl, the ionic conductivity increased to the maximum when the mass ratio was 10% (2.09 × 10^−4^ S cm^−1^). However, the ionic conductivity decreased slowly with the further increasing of the mass ratio. Figure 3E gives more visualized relationship of the ionic conductivity and the ASR as a function of Li_2.985_B_0.005_OCl mass ratio. The remarkable increased ionic conductivity from 0% to10% mass ratio corresponds to the evidently reduced grain boundary resistance, which is due to the compactly soldered LLZTO particles. The subsequently reduced ionic conductivity of CE is probably due to the relatively lower ionic conductivity of Li_2.985_B_0.005_OCl than that of LLZTO, which increased the grain boundary resistance of CE. It is also worth noticing that the bulk resistance of the CE kept increasing constantly, which is also due to the relatively lower ionic conductivity of Li_2.985_B_0.005_OCl.

It has been well known that the high electronic conductivity at the grain boundary of SSE is crucial for Li dendrite growth according to Wang's demonstration (Han et al., 2019). Consequently, reducing the electronic conductivity at the grain boundary will play an important role in Li dendrite suppression. Direct current polarization at different voltages from 0.4 to 0.8 V was tested to study the electronic conductivity of Li_2.985_B_0.005_ClO (Figure S6A) and LLZTO (Figure S6B), and the equilibrium current response in Figures S5A and S5B illustrated the smaller electronic conductivity of Li_2.985_B_0.005_ClO (5.2 × 10^−9^ S cm^−1^) than that of LLZTO (9.6 × 10^−8^ S cm^−1^). To evaluate the ability of Li dendrite suppression by reducing the electronic conductivity at the grain boundary, Li/LLZTO/Li and Li/CE/Li symmetric cells were tested under the direct current density stepping from 0.1 to 1.0 mA/cm^2^. Apart from the high electronic conductivity in the grain boundary, the interfacial resistance of the Li/electrolyte interface also plays an important role in Li dendrite growth. It is known that the Li/antiperovskite interfacial resistance is smaller than that of Li/garnet (Tian et al., 2018). Au modification layer was deposited to eliminate the difference on the Li/electrolyte interfacial resistance. Moreover, both Li/CE and Li/garnet interfaces could be further modified to promote the Li wettability by this Au modification layer. With the modification of Au interlayer, intimate contact of Li/LLZTO and Li/CE was obtained (Figures S7A and S7B). The two interfacial resistances were in the same level, which can be seen from the second arc in the EIS profile, and calculated to be 226 Ω cm^2^ for Li/LLZTO and 208 Ω cm^2^ for Li/CE (Figure S7C). The pristine LLZTO was sintered at 1,100°C to reduce the ionic transport resistance at the grain boundary, and a similar overall ionic conductivity was also obtained according to the first arc in the EIS profile. As is displayed in Figure 3F, the Li/LLZTO/Li symmetric cell displays a CCD of 0.6 mA/cm^2^, whereas the CCD of Li/CE/Li symmetric cell increased to 0.8 mA/cm^2^, indicating that the low electronic conductivity of Li_2.985_B_0.005_OCl at the grain boundary of LLZTO played an important role in Li dendrite suppression. After CCD test, the symmetric cells were dissected and Li dendrites can be found in the grain boundary in both LLZTO and CE (Figures S7D and S7E).

### Characterization of the Cathode/Electrolyte Interfaces

**Figure 4:**
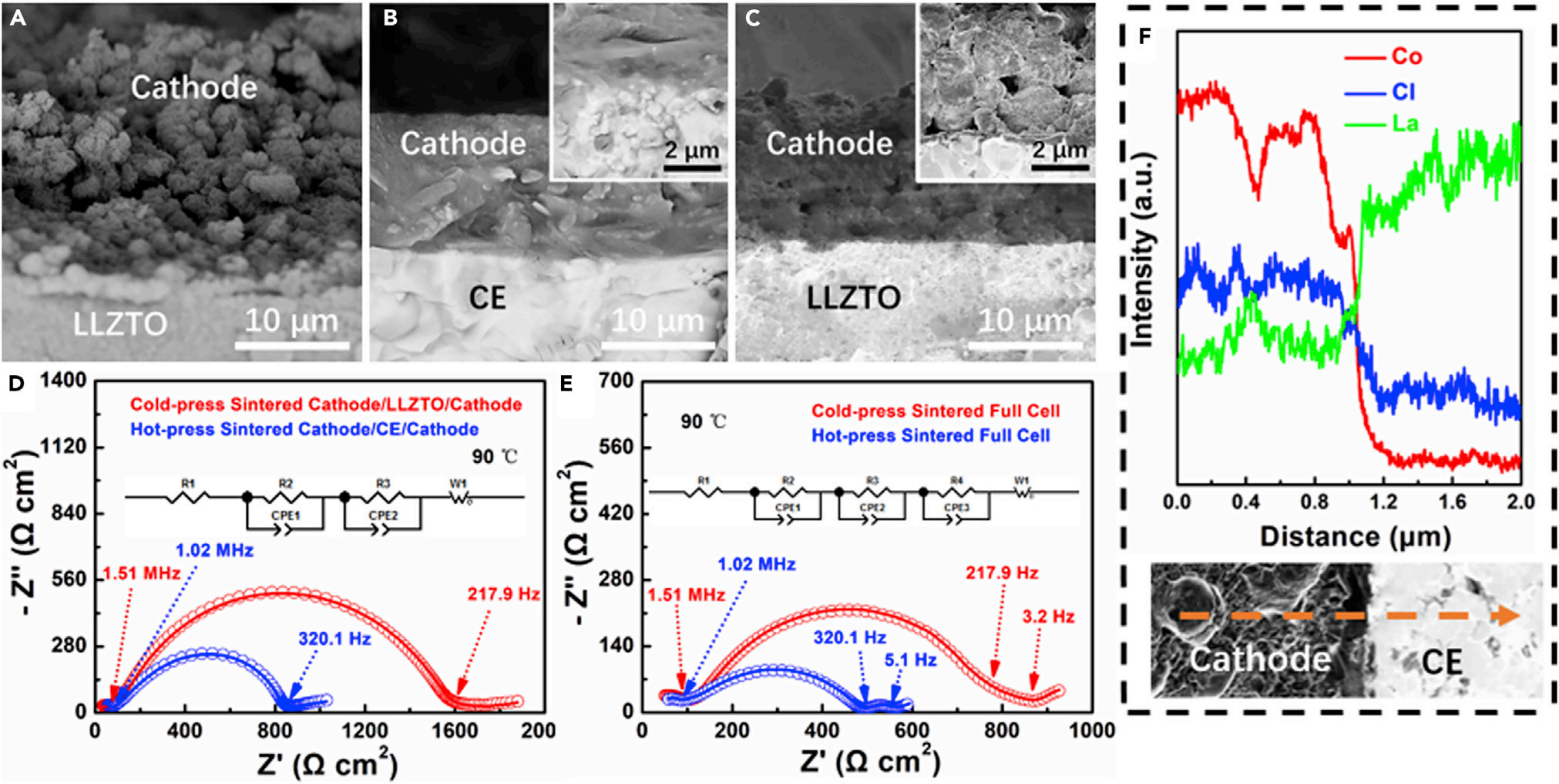
The Cathode/Electrolyte Interfaces Characterization (A–C) Cross-sectional SEM image of cathode/electrolyte interface (A) before and (B) after hot press and (C) after conventional cold-pressed sintering. (D) EIS profiles of the cold-press sintered and hot-press sintered cathode/electrolyte/cathode symmetric cells at 90°C. (E) EIS profiles of the cold-press sintered and hot-press sintered LCO/electrolyte/Li full cells at 90°C. (F) Elemental line scanning of the cathode/CE interfaces after hot pressing. The imaginary line corresponds to the scanning region.

The LCO@Li_2.985_B_0.005_OCl and CE were made into slurry and spin-coated on the pre-cold pressed CE pellet. As the cross-sectional SEM image demonstrated in Figure 4A, the as-coated cathode is porous. After being hot-pressed at 400°C, the cathode was soldered compactly and no voids can be found (Figure 4B). By contrast, the conventional cold-press sintered ACLB leaves a large sum of voids not only at the interface, but also inside the cathode (Figure 4C), which could lead to a larger impedance. To make a closer comparison of the hot-press sintered cathode/CE interface and the conventional cold-press sintered cathode/LLZTO interface, cathode/electrolyte/cathode symmetric cells were assembled and tested at 90°C. According to the second arcs in Figure 4D, the ASR of the cathode/LLZTO and the cathode/CE interfacial resistances are calculated to be 769 and 386 Ω cm^2^, indicating the superiority of the hot-pressing treatment by densifying the cathode and reducing the voids at the interface. Figure 4E displays the impedance of the LCO/electrolyte/Li full cells without and with hot pressing at 90°C. It is obvious that the conventional cold-press sintered ACLB possesses much huger impedance than the hot-pressed one, which is mainly due to the larger cathode/LLZTO interfacial resistance. Figure 4F illustrates the elemental line scanning of the cathode/CE interfaces. No mutual diffusion of Co and La at the interface can be indexed, indicating the successfully suppressed La_2_CoO_4_ phase through Li_2.985_B_0.005_OCl *in situ* coating and the low sintering temperature.

### Electrochemical Performance of the ACLBs with Different Solders

**Figure 5:**
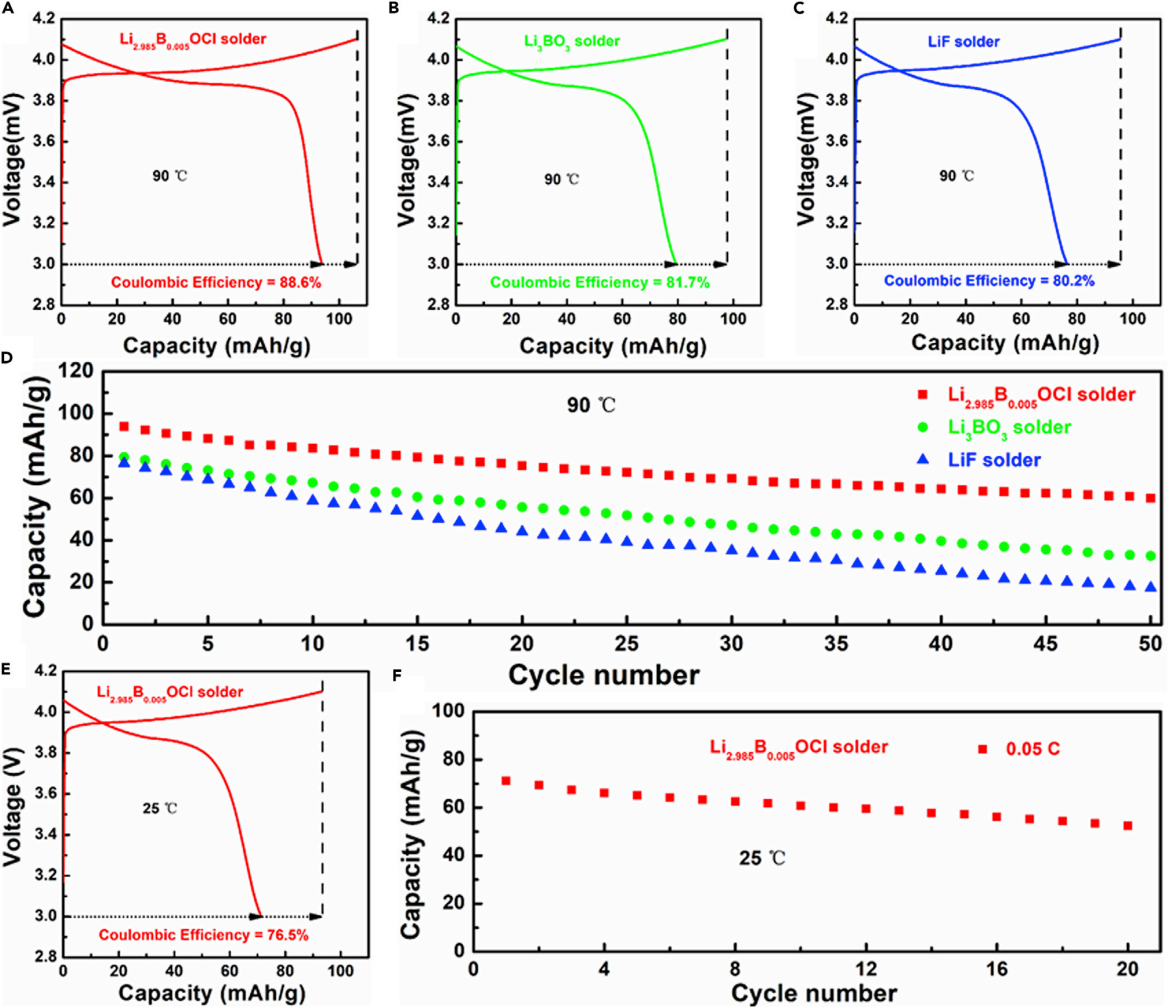
Electrochemical Performance of the Full Cells (A–C) Initial charge/discharge curves of the ACLBs at 90°C with (A) Li_2.985_B_0.005_OCl, (B) Li_3_BO_3_, and (C) LiF solders. (D) Cycling of the ACLBs. (E and F) (E) Initial charge/discharge curves, and (F) cycling of the Li_2.985_B_0.005_OCl soldered ACLBs at 25°C.

Three different kinds of ACLBs were assembled by hot pressing to evaluate the effects of the different solders on each ACLB. The sintering temperatures of Li_3_BO_3_ and LiF soldered ACLBs were adjusted to 700°C and 900°C, which were higher than each melting point. According to Ohsuku's research, there is a phase transition from rhombohedral to monoclinic in LCO at the open-circuit voltage of 4.12 V (Ohzuku and Ueda, 1994). Consequently, the charging cutoff potential was set to be 4.1 V (versus Li/Li^+^) to avoid the unwanted phase transition. Figures 5A–5C shows the initial charge/discharge curves of the ACLBs at 90°C with Li_2.985_B_0.005_OCl, Li_3_BO_3_, and LiF solders. Li_2.985_B_0.005_OCl soldered ACLB delivered the highest initial discharge capacity of 93.8 mAh/g and a coulombic efficiency of 88.6% (Figure 5A), whereas the Li_3_BO_3_ soldered counterpart displays a much smaller initial discharge capacity of 79.3 mAh/g and a coulombic efficiency of 81.7% (Figure 5B). In comparison, LiF soldered ACLB displays the lowest initial discharge capacity of 76.4 mAh/g, and the coulombic efficiency is also as low as 80.2% (Figure 5C). There are two main reasons that probably lead to the inferior electrochemical performance of the ACLB with Li_3_BO_3_ and LiF solders. First, as the EIS profiles shown in Figure S8, the ionic conductivities of two solders are too low to provide a satisfactory Li^+^ transportation environment, which are only 2.3 × 10^−6^ (Li_3_BO_3_) and 6.7 × 10^−7^ S cm^−1^ (LiF). Second, owing to their insolubility, Li_3_BO_3_ and LiF were coated on LCO and LLZTO by mechanical ball milling, which probably leads to an unthoroughly segregated LCO and LLZTO. The soldering temperature for the two ACLBs are high enough to form the highly ionic resistive La_2_CoO_4_ phase once there remains a direct contact between LCO and LLZTO, and then leads to the degradation of the LCO/LLZTO interfaces. As the cycling of the three ACLBs shown in Figure 5D, Li_2.985_B_0.005_OCl soldered ACLB displays the most stable performance of 50 cycles, whereas the Li_3_BO_3_ and LiF show not only lower discharge capacities but also faster degradation.

Assembly information and electrochemical performances of the garnet-based ACLBs with different solders are summarized in Table S2. It is obvious that Li_2.985_B_0.005_OCl displays the highest ionic conductivity. Moreover, the Li_2.985_B_0.005_OCl soldered ACLB demonstrates the lowest sintering temperature, the highest initial coulombic efficiency, and the longest cycle life. The improved coulombic efficiency is probably because there contains no organic binder like ethyl cellulose, as the decomposition of organolithium compounds that were produced during the sintering is responsible for the low initial coulombic efficiency in the conventional garnet-based ACLBs Ohta et al., 2014. The Li_2.985_B_0.005_OCl soldered ACLB was also cycled in 25°C to evaluate its room temperature performance. But according to the initial charge/discharge curves and the cycling performance in Figures 5E and 5F, the ACLB demonstrates a smaller initial discharge capacity (71.3 mAh/g) and a faster degradation than that was cycled at 90°C. The phenomenon is mainly due to the larger room temperature impedance, which is about 5-fold increased than that at 90°C (Figure S9).

### *In Situ* EIS Profiles of the ACLBs at 90°C

**Figure 6:**
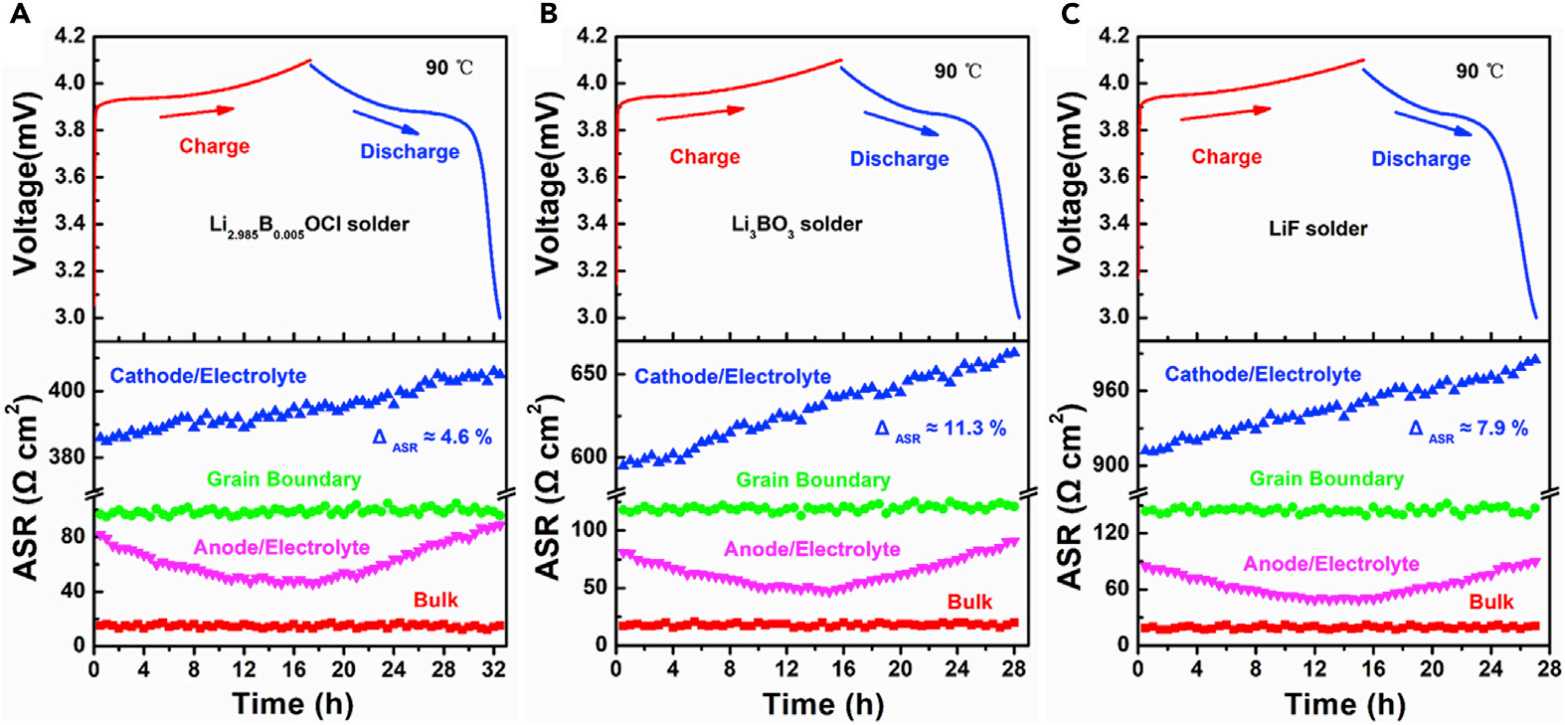
*In Situ* EIS Profiles of the ACLBs at 90°C with Different Solders during the Initial Cycle (A) Li_2.985_B_0.005_OCl, (B) Li_3_BO_3_, and (C) LiF.

For a more detailed investigation on the cycling stabilities, *in situ* impedances of the three kinds of ACLBs during the initial cycles have been tested and analyzed. As illustrated in Figures 6A–6C, the bulk and grain boundary resistances of each electrolyte show negligible changes, indicating that the solders are highly stable during the galvanostatic charge and discharge. With the decreasing of the ionic conductivity of the solders, the ASR of the grain boundary increased slightly, which are about 98 Ω cm^2^ for Li_2.985_B_0.005_OCl, 116 Ω cm^2^ for Li_3_BO_3_, and 143 Ω cm^2^ for LiF. It is interesting to find that all of the anode/electrolyte interfacial resistances display an evident decrease during charge and increase during discharge. The initial decreasing can be explained as the vanished interfacial holes or voids that were filled by the platted Li, which provided more intimate contact between Li and the electrolytes. The subsequent impedance augment can be comprehended as the interfacial gaps that were created by the stripped Li (Yang et al., 2018).

Moreover, The ASR of the cathode/electrolyte interface increased with the decreasing of the ionic conductivity of the solders, which are about 386 Ω cm^2^ for Li_2.985_B_0.005_OCl, 595 Ω cm^2^ for Li_3_BO_3_, and 912 Ω cm^2^ for LiF before cycling. It is also worth noticing that all of the cathode/electrolyte interfacial resistances increased to some extent after the initial cycle, which are 4.6%, 11.3%, and 7.9% for Li_2.985_B_0.005_OCl, Li_3_BO_3_, and LiF, respectively. Since it is known that the length of the c-axis and the a-axis in LCO changes conversely during charge/discharge, Asano et al., 2018 due to the rigid solid to solid contacts between LCO and the solders, the ASR augments can be explained as the Griffith cracks that were generated by the strain/stress of the LCO during charge/discharge. But according to the elasticity modulus of the solders in Table S3, Li_2.985_B_0.005_OCl displays the smallest elasticity modulus (7.8 GPa). In other words, Li_2.985_B_0.005_OCl is soft enough to release more strain/stress effects of the LCO than that of Li_3_BO_3_ and LiF, which resulted in the smaller ASR augment, higher initial coulombic efficiency, and more stable cycling. Comparatively, Li_3_BO_3_ and LiF show higher elasticity modulus of 20.5 and 11.9 GPa, which correspond with each ASR augment. The stiffer nature of the solders will lead to more cracks at the interfaces and deteriorate the cycling stabilities.

### Conclusion

Li_2.985_B_0.005_OCl anti-perovskite electrolyte was applied as sintering solder for garnet-based bulk-type ACLB, whose conductivity is as high as 6.8 × 10^−5^ S cm^−1^ and melting point is as low as 267°C. A novel alkaline aqueous solution-based *in situ* coating process was introduced to thoroughly segregate the LLZTO and LCO. Hot-press sintering was introduced to densify both CE and the cathode, and a low cathode/electrolyte ASR of 386 Ω cm^2^ was realized. Attributing to the smaller elasticity modulus, Li_2.985_B_0.005_OCl is also soft enough to release more strain/stress effects than the Li_3_BO_3_ and LiF counterparts, leading to a higher initial coulombic efficiency and more stable cycling. In addition, Li dendrite can be suppressed by the reduced electronic conductivity at the grain boundary. With the approaches that turned out to be effective, we believe that the improvements of the garnet-based bulk-type ACLB can be realized by integrating a solder with higher ionic conductivity, lower melting point, and smaller elasticity modulus.

### Limitations of the Study

The garnet-based all-ceramic lithium battery is very fragile after hot pressing, which needs to be very carefully held during the electrochemical characterization. Moreover, the active material loading is still very low. Enhancing the loading of the composited cathode will lead to higher interfacial resistance and deteriorates the cycling stability. Electronic conductors such as carbon nano tubes (CNTs) or Super-P need to be added once the cathode loading is increased.

## Methods

All methods can be found in the accompanying Transparent Methods supplemental file.

## Data and Code Availability

All data, models, and code generated or used during the study appear in the submitted article.

## Supplemental Information

Document S1. Transparent Methods, Figures S1–S9, and Tables S1–S3
