# Supplementary material for: Garnet-Based All-Ceramic Lithium Battery Enabled by Li2.985B0.005OCl Solder
Source: iScience. 2020 Apr 18;23(5):101071. doi: 10.1016/j.isci.2020.101071 (PMC7195548; doi:10.1016/j.isci.2020.101071)
Supplement: Document S1. Transparent Methods, Figures S1–S9, and Tables S1–S3 [file mmc1.pdf]

iScience, Volume *23*

Supplemental Information

Garnet-Based All-Ceramic Lithium Battery Enabled by $Li_{2.985}B_{0.005}OCl$ Solder

Wuliang Feng, Zhengzhe Lai, Xiaoli Dong, Panlong Li, Yonggang Wang, and Yongyao Xia

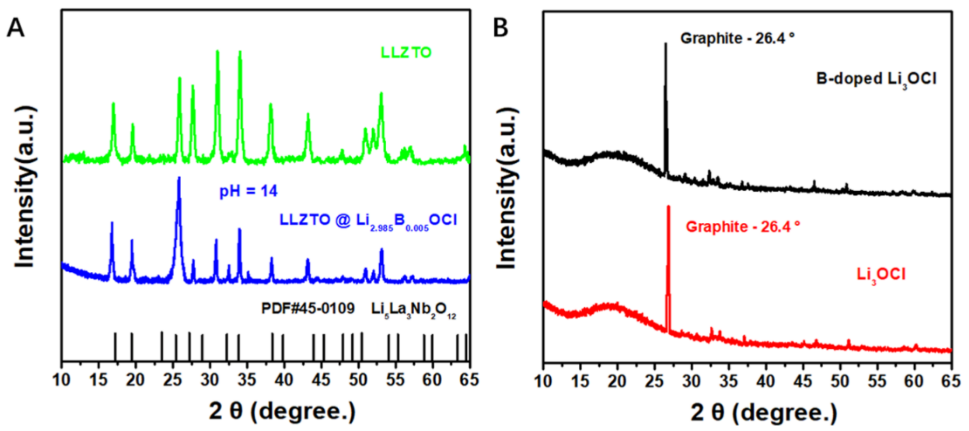

Figure S1. XRD pattern of the doped and undoped anti-perovskites, pristine LLZTO and the coated LLZTO, related to Figure 1.

(A) The pristine LLZTO and the in-situ coated LLZTO.

(B) The doped and undoped Li_3OCl.

A tiny amount of graphite was mixed in the anti-perovskites to adjust the spectral shifts due to the test error between the doped and undoped Li_3ClO.

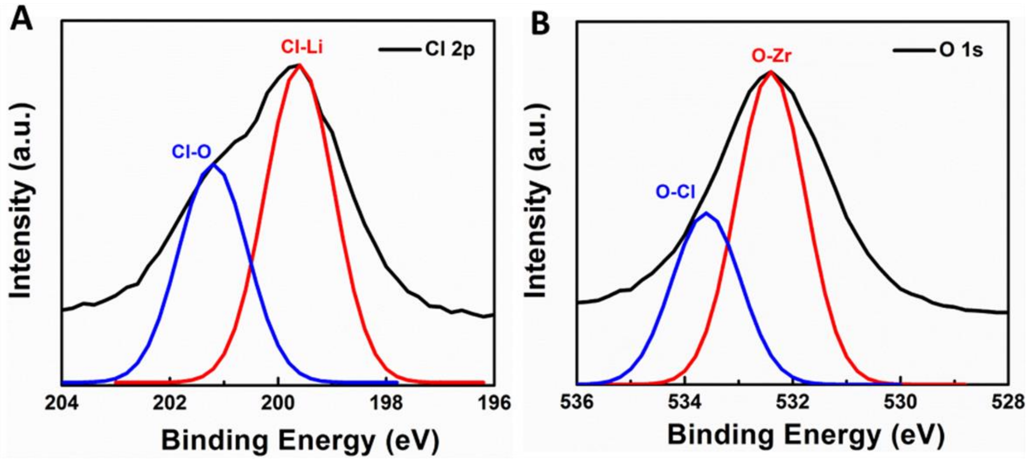

Figure S2. XPS spectra of $Li_{2.985}B_{0.005}OCl$ coated LLZTO, related to Figure 2.

(C) The core-level of Cl 2p.

(D) The core-level of O 1s.

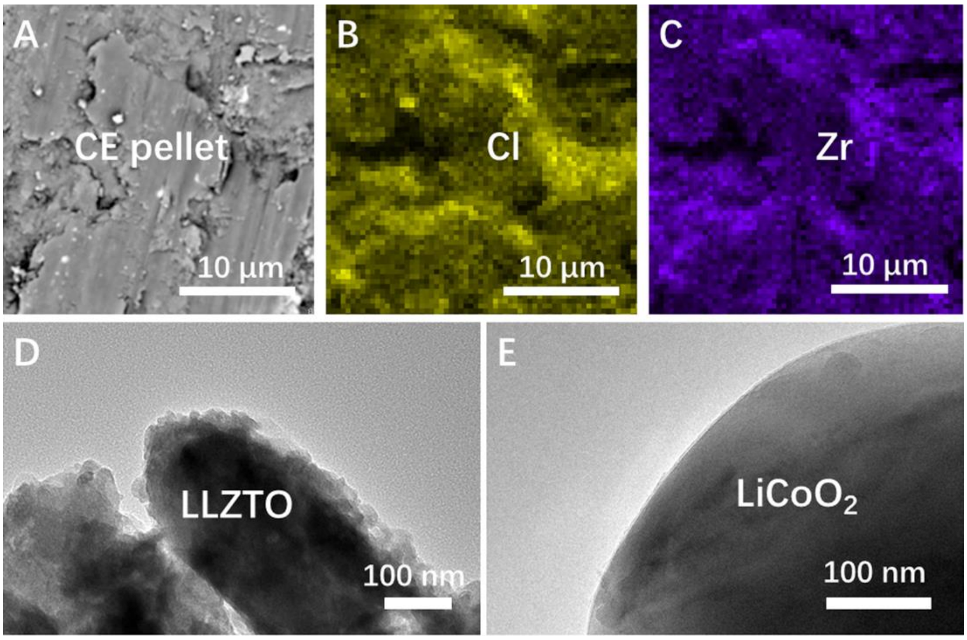

Figure S3. Mapping of the composited electrolyte, TEM image of the pristine LLZTO and the LCO particles, related to Figure 3.

(A) SEM image of the surface of the CE pellet.

(B) Mapping of Cl.

(C) Mapping of Zr.

(D) TEM image of the pristine LLZTO.

(E) TEM image of the LCO particles.

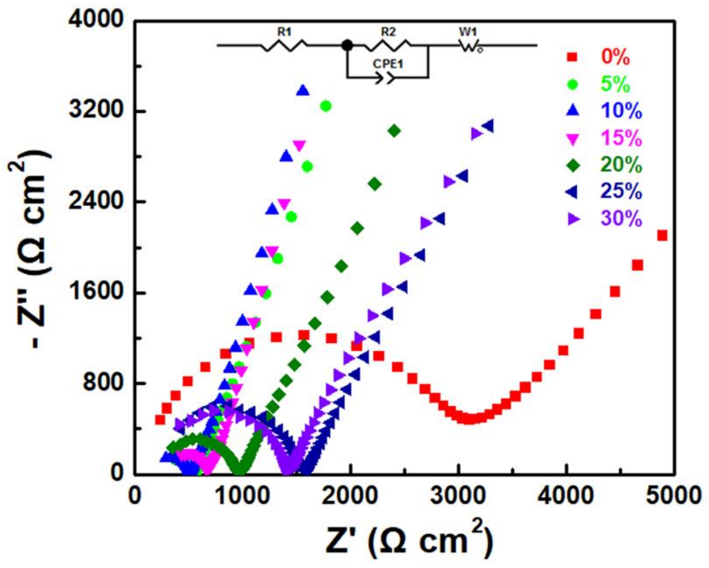

Figure S4. EIS profiles of the CE with different $Li_{2.985}B_{0.005}OCl$ mass ratios, related to Figure 3.

Table S1. Fitting results of the EIS profiles of the CE with different $Li_{2.985}B_{0.005}OCl$ mass ratios, related to Figure 3.

$Li_{2.985}B_{0.005}OCl$ Mass Ratio (%)	ASR_{bulk} (Ω cm^2)	$ASR_{grain\ boundary}$ (Ω cm^2)	Ionic Conductivity (S cm^{-1})
0	53	3092	0.31×10^{-4}
5	55	471	1.89×10^{-4}
10	63	426	2.09×10^{-4}
15	81	511	1.69×10^{-4}
20	102	889	1.01×10^{-4}
25	138	1243	0.73×10^{-4}
30	165	1429	0.64×10^{-4}

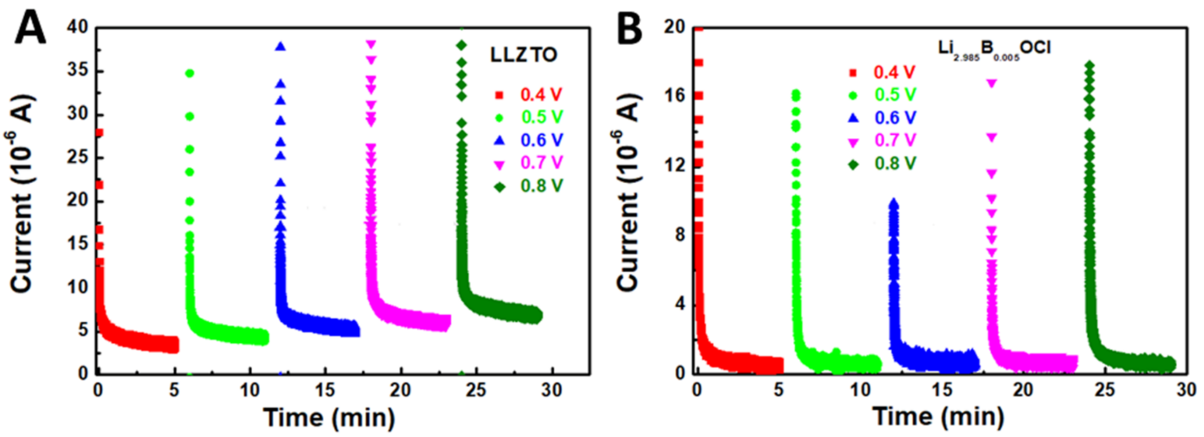

Figure S5. Direct current polarization curves of LLZTO and $Li_{2.985}B_{0.005}OCl$, related to Figure 3.

(A) LLZTO at different voltages from 0.4 to 0.8 V.

(B) $Li_{2.985}B_{0.005}OCl$ at different voltages from 0.4 to 0.8 V.

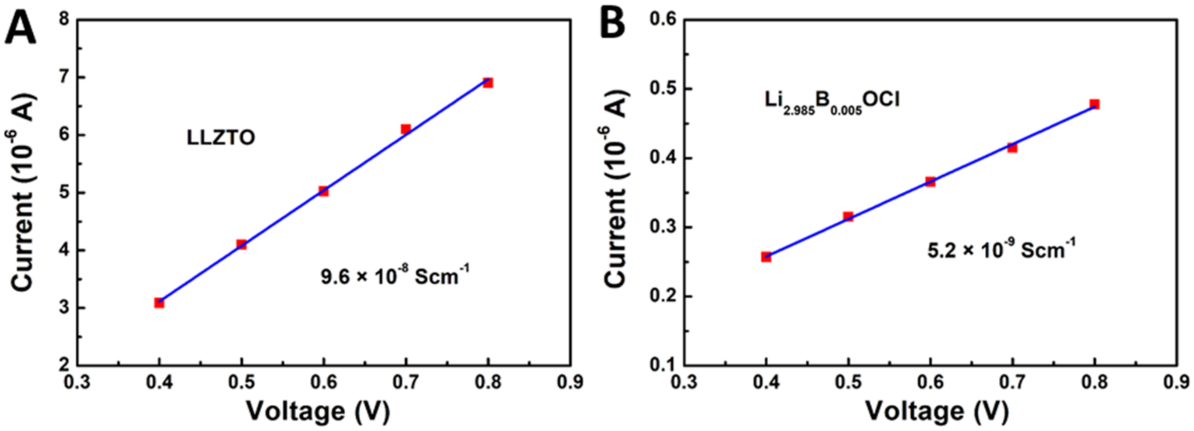

Figure S6. Equilibrium current response of LLZTO and $Li_{2.985}B_{0.005}OCl$, related to Figure 3.

(A) LLZTO at different voltages from 0.4 to 0.8 V.

(B) $Li_{2.985}B_{0.005}OCl$ at different voltages from 0.4 to 0.8 V.

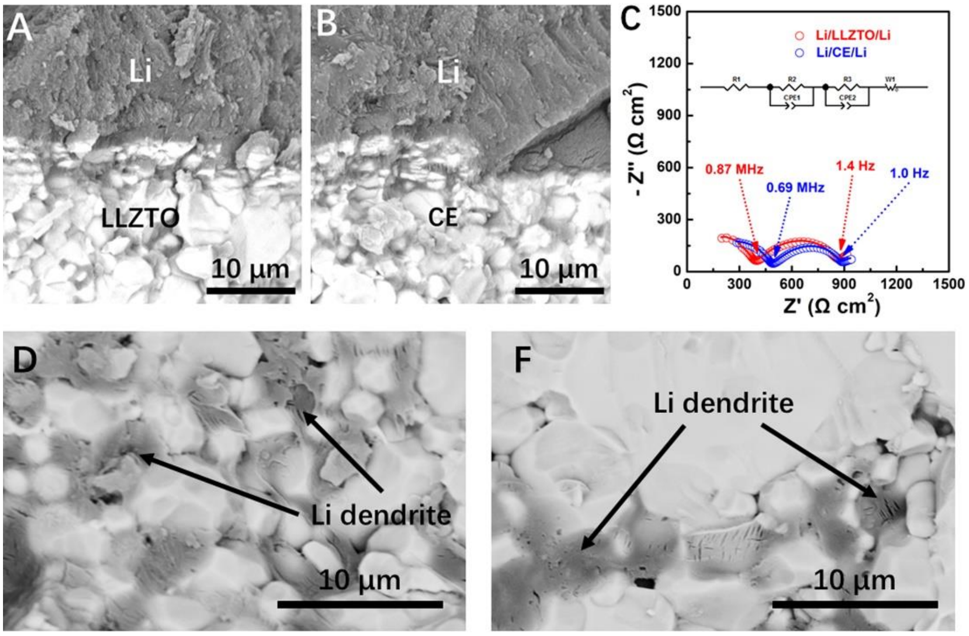

Figure S7. Cross sectional SEM image, EIS profile of the Li/LLZTO/Li and Li/CE/Li symmetric cells, and the Li dendrites characterization, related to Figure 3.

(A) Cross sectional SEM image of Li/LLZTO interface.

(B) Cross sectional SEM image Li/CE interface.

(C) EIS profiles of the Li/LLZTO/Li and Li/CE/Li symmetric cells.

(D) Li dendrites characterization after CCD test, Li/LLZTO/Li.

(F) Li dendrites characterization after CCD test, Li/CE/Li.

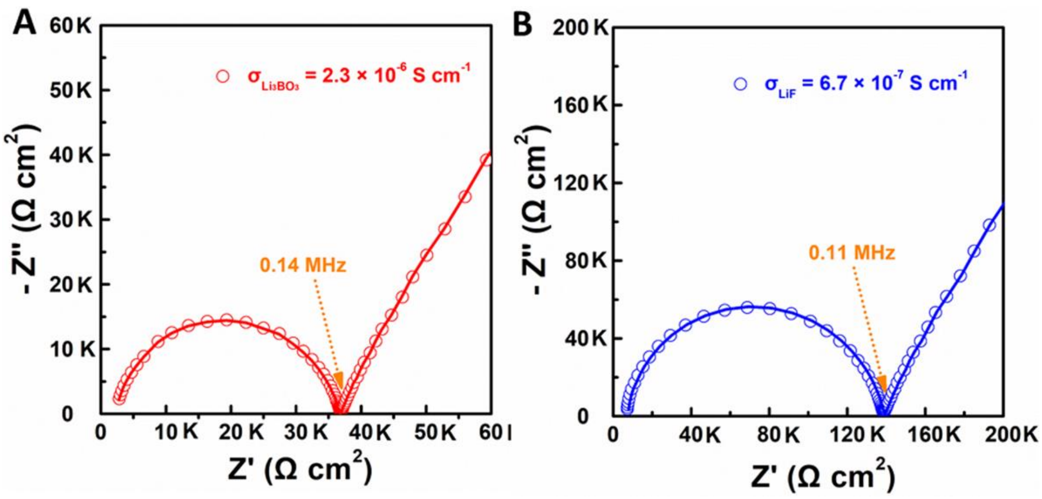

Figure S8. EIS profiles of the Li_3BO_3 and LiF solders, **related to Figure 5.**

(A) EIS profile of Li_3BO_3.

(B) EIS profile of LiF.

Table S2. Assembly information and the electrochemical performance of the garnet-based bulk-type ACLBs, related to Figure 5.

References	Solder	Li^+ Conductivity	Sintering Temperature	Testing Temperature	Current	Initial Coulombic Efficiency	Cycles
Park et al., 2016	Li_3BO_3	2×10^{-6} Scm^{-1}	700 °C	50 °C	0.2 C	54.7 %	10
Okumura et al., 2016	$Li_{2.2}C_{0.8}B_{0.2}O_3$	$7.3\times10^{-5}Scm^{-1}$, 120 °C	660 °C	120 °C	32 μA/cm^2	80.8 %	20
Ohta et al., 2013	Li_3BO_3	2×10^{-6} Scm^{-1}	700 °C	25 °C	0.05 C	~ 85 %	5
Ohta et al., 2014	Li_3BO_3	$2\times10^{-6}Scm^{-1}$	790 °C	25 °C	0.01 C	79.6 %	1
Han et al., 2018	$Li_{2.3}C_{0.7}B_{0.3}O_3$	~ $10^{-5}Scm^{-1}$, 100 °C	700 °C	100 / 25 °C	0.05 C	~ 74.6 %	40
Liu et al., 2018	$Li_3BO_3.In_2SnO_5$	---	600-700 °C	80 °C,	10-20 μA/cm^2	~ 60 %	5
This work	$Li_{2.985}B_{0.005}OCl$	$6.8\times10^{-5}Scm^{-1}$	400 °C	90 °C	0.05 C	88.6 %	50

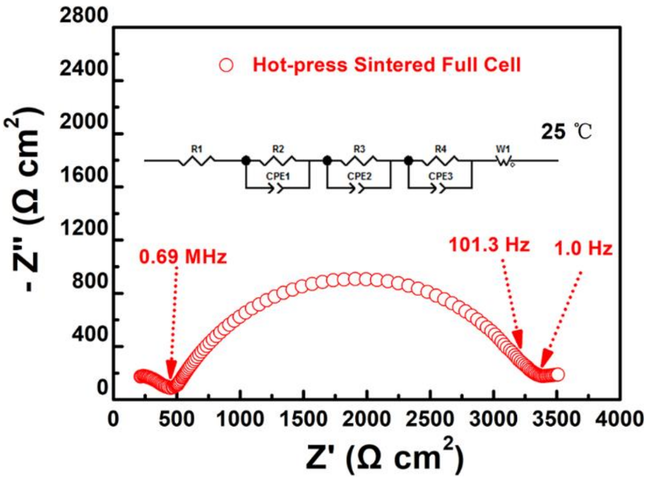

Figure S9. EIS profile of the hot-press sintered LCO/CE/Li full cells at 25 °C, related to Figure 5.

Table S3. Elasticity Modulus of the three kinds of solders, related to Figure 6.

---	$Li_{2.985}B_{0.005}OCl$	Li_3BO_3	LiF
Elasticity Modulus/GPa	7.8	20.5	11.9

Transparent Methods

In-situ coating of LLZTO@$Li_{2.985}B_{0.005}OCl$ and LCO@$Li_{2.985}B_{0.005}OCl$. LLZTO powder was purchased from Kejing material technology co. LTD. In-situ coating of $Li_{2.985}B_{0.005}OCl$ includes the dissolution of the stoichiometric amounts of LiCl, LiOH and H_3BO_3 in deionized water. The pH value of the solution was adjusted to 14 by controlling the concentration of LiOH (> 1 M). LLZTO and LCO were added into the strong basicity solution separately, then dried under agitation. The as prepared powder was sintered at 350 °C for 5 h under vacuum and then grounded in glovebox. The mass ratio of $Li_{2.985}B_{0.005}OCl$ was controlled by adjusting the concentration of LiCl, LiOH and H_3BO_3.

Assembly of Li/LLZTO/Li and Li/CE/Li symmetric cells. The pristine LLZTO pellet was made by cold pressing under the pressure of 300 MPa and sintered at 1200 °C for 12 h with the coverage of the same mother powder. The CE pellet was made by pre-pressing under the pressure of 30 MPa in room temperature, then hot-pressed at 400 °C under 50 MPa for 1 h. A 20 nm Au modification layer was deposited on LLZTO and CE pellets by magnetron sputtering. Li foils were attached on the LLZTO and CE pellets and heated at 200 °C for 30 min under the pressure of about 5000 Pa.

Hot-pressing of the ACLBs. The cathode consists of $LiCoO_2$ active material, LLZTO electrolyte and $Li_{2.985}B_{0.005}OCl$ solder (mass ratio = 11 : 5 : 4). Methylpyrrolidone (NMP) was added into the powder and the slurry was spin-coated on the pre-pressed CE pellet. The NMP solvent was evaporated under 80 °C and the active material loading was about 0.8 mg. The cathode was casted in a 10 mm round area and the mass ratio of the active material is 55 %, so the cathode loading can be calculated as 1.85 mg/cm^2. It should be noticed that the specific

surface area of the most of the electronic conductors are huge. The participation of the electronic conductivities will reduce the contact area of the active material with the ionic conductor, and the transportation of Li^+ will be severely restricted. Moreover, the thickness of the cathode was very thin, which had already provided an applicable electronic conductivity for the full cell. The ACLB was prepared by hot-pressing the cathode and the CE together at 400 °C for 1 h under the pressure of 50 MPa. The contrast sample, Li_3BO_3 solder were synthesized through solid reaction (ball milling the mixture of Li_2CO_3 and B_2O_3, then sintered at 600 °C for 8 h) and LiF was purchased from Alfa Aesar. Li_3BO_3 and LiF were coated on LLZTO and $LiCoO_2$ by ball milling. The mass ratio of $LiCoO_2$, LLZTO and solders were also 11 : 5 : 4, and the hot-pressing temperature were increased to 700 °C for Li_3BO_3 and 900 °C for LiF soldered counterparts.

Characterization. X-ray diffraction (XRD) data was characterized by Bruker D8 advance diffractometer by Cu Kα radiation. X-ray photoelectron spectroscopy (XPS) was carried out on RBD upgraded PHI-5000C ESCA system (PerkinElmer) with Mg Kα radiation (hν = 1253.6 eV). The structural changes, melting and solidification points of the anti-perovskites were obtained through differential scanning calorimetry (DSC, NETZSCH, Germany). The morphology of the cross sectional LLZTO and CE pellets and electrode/electrolyte interface were characterized by transmission electron microscopy (TEM, Joel JEM2010) and scanning electron microscopy (SEM, Joel JSM6390). The impedance of the electrolyte, symmetric cells and the ACLBs were measured by electrochemical impedance spectroscopy (EIS) with a frequency range from 7 MHz to 1 Hz with 50 mV perturbation amplitude. Galvanostatic stripping and plating of the symmetric cell, cyclic properties were operated on LAND

CT2001A Battery Cycler (Wuhan, China).

REFERENCES

Park, K., Yu, B.C., Jung, J.W., Li, Y.T., Zhou, W.D., Gao, H.C., Son, S., Goodenough, J.B. (2016). Electrochemical Nature of the Cathode Interface for a Solid-State Lithium-Ion Battery: Interface between $LiCoO_2$ and Garnet $Li_7La_3Zr_2O_{12}$. Chem. Mater. *28*, 8051-8059.

Okumura, T., Takeuchi, T. & Kobayashi, H. (2016). All-solid-state lithium-ion battery using $Li_{2.2}C_{0.8}B_{0.2}O_3$ electrolyte. Solid State Ionics *288*, 248-252.

Ohta, S., Komagata, S., Seki, J., Saeki, T., Morishita, S. & Asaoka, T. (2013). All-solid-state lithium ion battery using garnet-type oxide and Li_3BO_3 solid electrolytes fabricated by screen-printing. J. Power Sources *238*, 53-56.

Ohta, S., Seki, J., Yagi, Y., Kihira, Y., Tani, T. & Asaoka, T. (2014). Co-sinterable lithium garnet-type oxide electrolyte with cathode for all-solid-state lithium ion battery. J. Power Sources *265*, 40-44.

Han, F., Yue, J., Chen, C., Zhao, N., Fan, X.L., Ma, Z.H., Gao, T., Wang, F., Guo, X.X., Wang, C.S. (2018). Interphase Engineering Enabled All-Ceramic Lithium Battery. Joule *2*, 497-508.

Liu, T., Zhang, Y.B., Zhang, X., Wang, L., Zhao, S.X., Lin, Y.H., Shen, Y., Luo, J., Li, L.L., Nan, C.W. (2018). Enhanced electrochemical performance of bulk type oxide ceramic lithium batteries enabled by interface modification. J. Mater. Chem. A *6*, 4649–4657.
